# Nonlinear Vibration of a Pre-Stressed Water-Filled Single-Walled Carbon Nanotube Using Shell Model

**DOI:** 10.3390/nano10050974

**Published:** 2020-05-18

**Authors:** Mahmoud Mohamed Selim, Awad Musa

**Affiliations:** 1 Department of Mathematics, College of Science and Humanities in Al-Aflaj, Prince Sattam bin Abdul-Aziz University, Al-Aflaj 11912, Saudi Arabia; 2 Department of Mathematics, Suez Faculty of Science, Suez University, Suez 34891, Egypt; 3 Department of Physics, College of Science and Humanities in Al-Aflaj, Prince Sattam bin Abdul-Aziz University, Al-Aflaj 11912, Saudi Arabia; a.tinebour@psau.edu.sa; 4 Department of Physics, College of Science, SUST, Khartoum 678 73, Sudan

**Keywords:** nonlinear vibration, Donnell’s shell model, initial stresses, water-filled, single-wall carbon nanotube (SWCNT)

## Abstract

This paper is an attempt to study the nonlinear vibration of a pre-stressed single-walled carbon nanotube (SWCNT) with water-filled and simply supported ends. A new analytical formula is obtained for the nonlinear model based on the simplified Donnell’s shell theory. The effects of internal fluid on the coupling vibration of the SWCNT–water system are discussed in detail. Furthermore, the influence of the different nanotube thicknesses and radiuses on the nonlinear vibration frequencies is investigated according to the shell theory. Numerical calculations are done to show the effectiveness of the proposed schemes. The results show that the nonlinear frequency grew with the increasing nonlinear parameters (radius and thickness of nanotube). In addition, it is shown that the influence of the nonlinear parameters is greater at the lower mode in comparison with the higher mode for the same nanotube thickness and radius.

## 1. Introduction

Carbon nanotubes have become one of the most important nanomaterials for nanotechnology; they have distinguished mechanical and electrical properties, and have had notable applications in nanodevices in recent years [1,2,3,4,5,6,7]. To understand the dynamic behavior of carbon nanotubes, numerous researchers have conducted computational simulations to study the vibration and wave propagation in carbon nanotubes [8,9,10,11]. Recently, beam and cylindrical shell models have been used to study the bending, buckling and vibrational behaviors of carbon nanotubes [12]. Using Donnell’s shell equations, Sun and Liu [13] have studied the free vibration of multi-walled carbon nanotubes (MWCNTs). Asghar et al. [14] have studied the non-local effect on the vibration analysis of double-walled carbon nanotubes based on Donnell’s shell theory. Yan et al. [15] have investigated the free vibration of conical shell structures reinforced by graphene platelets (GPLs) and the elastic properties of the nanocomposite have been obtained by employing the shell model based on Donnell’s shell theory. Zhang et al. [16] has studied the critical buckling strains of axial loads using both the beam and cylindrical shell model. To know and take the necessary steps to control the structural vibration response of nanodevices, nonlinear vibration analysis has become very important in designing the structure of such nanodevices. The large amplitude (non-linear vibration) of carbon nanotubes, due to the effects of the large deformation within the elastic limit on the carbon nanotubes, has received considerable attention. Yan et al. [17] have modeled the nonlinear free vibration of double-walled carbon nanotubes using Donnell’s shell theory. Nowadays, the transport properties of water confined in one-dimensional nanochannels are of great interest in physics and medicine. An ideal model for these studies is water that is confined inside carbon nanotubes—specifically, single-walled carbon nanotubes [18,19,20,21,22,23]. However, there is little systematic consideration concerning the initial stress effects in water-filled CNTs in the literature. On the other hand, CNTs often suffer from initial stresses due to residual stress, thermal effects, surface effects, mismatches between the material properties of CNTs and surrounding mediums, initial external loads and other physical issues. In this field, the effects of initial stress on the non-coaxial resonance of multi-walled nanotubes (MWNTs) have been investigated by the theories of Euler–Bernoulli and Timoshenko beams, respectively, in Wang et al. [24] and Cai et al. [25]. Based on the Euler–Bernoulli beam theory, Zhang et al. [26] studied the transverse vibrations of DWNTs under compressive axial load. They pointed out that the natural frequencies are dependent on the axial load and decrease with an increase in the axial load, and that the associated amplitude ratios of the inner to the outer tubes of DWNTs are independent of the axial load. Lu et al. [27] adopted a nonlocal Euler–Bernoulli beam model to analyze the wave and vibration characteristics of one-dimensional (1D) nanostructures with initial axial stress. Furthermore, Wang et al. [28] used a nonlocal Timoshenko beam model to deal with the free vibration of micro- and nanobeams with initial stress. The vibration of multi-wall carbon nanotubes (MWCNTs) and the wave propagation of double-wall carbon nanotubes (DWCNTs) have been studied based on the Flügge shell equation [29,30]. Selim [31,32,33] demonstrated how to construct and analyze the propagation of dilatation and transverse waves in a pre-stressed plate and single-wall carbon nanotube using local and nonlocal scale effects.

In this work, the nonlinear vibration of an initially stressed water-filled single-walled carbon nanotube is investigated using shell theory. Furthermore, the influence of the different nanotube thicknesses and radiuses on the nonlinear vibration frequencies is investigated according to Donnell’s shell theory. Numerical calculations are done and shown graphically.

## 2. Formulation of the Problem

**Figure 1:**
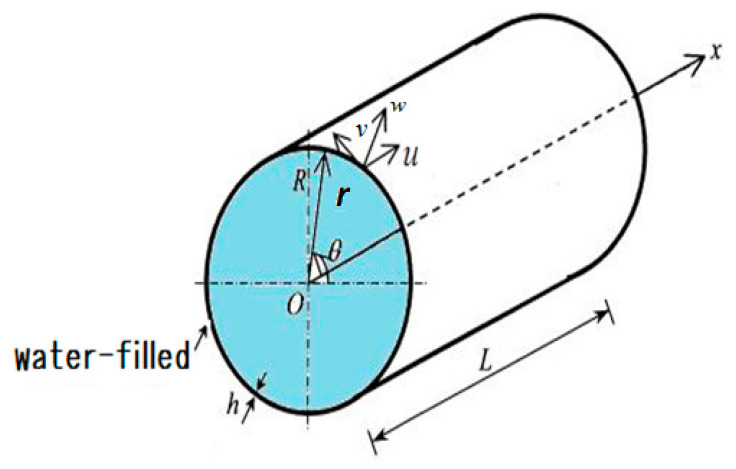
Tube geometry and coordinate system used.

Assuming small strains and displacements, and considering the thin shell theory, Figure 1 illustrates the cylindrical coordinate system (x,θ,r) and the geometry of the model. *u*, *v* and *w* are the axial, circumferential and radial displacements, respectively.

The nonlinear shallow-shell equations of motion based on Donnell’s theory are given by Amabili [34]:

(1) $$D\nabla^{4}w_{t}+\rho_{t}\frac{\partial^{2}w_{t}}{\partial t^{2}}=\frac{1}{R_{t}}\frac{\partial^{2}F}{\partial x^{2}},$$

(2) $$\eta \frac{\partial^{2}w_{s}}{\partial x^{2}}-\zeta_{s}=\rho_{s}\frac{\partial^{2}w_{s}}{\partial t^{2}},$$

(3) $$\nabla^{4}F=-\frac{Eh}{R_{t}}\frac{\partial^{2}w_{t}}{\partial x^{2}},$$

(4) $$\nabla^{4}={\left[\frac{\partial^{2}}{\partial x^{2}}-\frac{1}{R_{t}{}^{2}}\frac{\partial^{2}}{\partial \theta^{2}}\right]}^{2},$$

(5) $$\zeta_{s}=Q_{s}-P\frac{\partial^{2}w_{t}}{\partial x^{2}},$$

(6) $$Q_{s}=\rho_{s}(\frac{L}{m\pi })\frac{I_{n}(\tau_{s})}{{I^{\prime }}_{n}(\tau_{s})}\frac{\partial^{2}w_{s}}{\partial t^{2}},$$

(7) $$\tau_{s}=\frac{m\pi R_{s}}{L},$$

where $R_{t}$ is the carbon nanotube radius, t is the time, $w_{t}$ is the radial displacement, $\rho_{t}$ is the density, *h* is the tube thickness, L is the length of nanotube, E is the Young’s modulus, $D=\frac{Eh^{3}}{12(1-\nu^{2})}$ is the bending stiffness and ν is the Poisson ratio.

For the water shell, $\rho_{s}$ is density, $Q_{s}$ is the flow pressure, $R_{s}$ is the radius, $w_{s}$ is the radial displacement, η is the carbon nanotube–water surface tension, P is the initial compression stress, $I_{n}$ and ${I^{\prime }}_{n}$ are the modified Bessel function of order *n* and its first derivative with respect to the argument and F is the unknown stress function, which will be determined. For the present study, the displacements, slope, moments, shears, and stresses must all satisfy the continuity conditions:

(8) $$w_{t}(x,\theta ,t)=w_{t}(x,\theta +2\pi R,t),$$

(9) $$w_{s}(x,\theta ,t)=w_{s}(x,\theta +2\pi R,t).$$

## 3. Solution of the Problem

An approximate solution will be used to solve Equations (1) and (2). First, we choose a vibration mode for w and solving Equation (2). Galerkin’s method will be used in Equation (1) to find F. We should examine w and F throughout the calculations to verify the necessary continuity requirements of Equation (3). By choosing the vibration modes of nonlinear vibrations, the nonlinear versions of Equations (1) and (2) have the following solutions [35]:

(10) $$w_{t}=\sum_{m=1}^{2}\Omega_{m,n}(t)\sin(m\pi x/L)\cos(n\theta )+\frac{n^{2}}{4R_{t}}\Omega_{m,n}{\left(t\right)}^{2}\sin{\left(m\pi x/L\right)}^{2},$$

(11) $$w_{s}=\sum_{m=1}^{2}\Omega_{m+2,n}(t)\sin(m\pi x/L)\cos(n\theta )+\frac{n^{2}}{4R_{s}}\Omega_{m,n}{\left(t\right)}^{2}\sin{\left(m\pi x/L\right)}^{2}$$

where $\Omega_{m,n}(t)$ is the unknown function of time m is the $m^{th}$ axial mode and *n* is the $n^{th}$ circumferential mode. Equations (10) and (11) represent the deflection modes assumed in the present problem.

### 3.1. Application of Galerkin’s Method

The particular solution of the function F is determined by substituting Equations (10) and (11) into Equation (2), giving us

(12) $$F_{p}=Eh\left[\sum_{m=1}^{2}\left(c_{1}\Omega_{m,n}(t)\sin(m\pi x/L)\cos(n\theta )+c_{2}\Omega^{2}{}_{m,n}(t)\cos(2n\theta )+c_{3}\Omega^{3}{}_{m,n}(t)\sin(3m\pi x/L)\cos(n\theta )\right)\right],$$

where

(13) $$c_{1}=\frac{Ehm^{2}\pi^{2}L^{2}}{\left(m^{4}\pi^{4}+2m^{2}\pi^{2}n^{2}L^{2}+n^{4}L^{4}\right)},\;c_{2}=-\frac{Ehm^{2}\pi^{2}}{32n^{2}L^{2}},c_{3}=\frac{Ehm^{2}\pi^{2}n^{4}L^{2}}{4}\left(\frac{1}{{\left(9m^{2}\pi^{2}+n^{2}L^{2}\right)}^{2}}-\frac{1}{{\left(m^{2}\pi^{2}+n^{2}L^{2}\right)}^{2}}\right).$$

To solve Equation (1), we will substitute Equations (10)–(12) into Equation (1). Galerkin’s procedure provides a very powerful approximation method by employing any set of basic functions φ, which transform a system of nonlinear partial differential equations to a solvable system of nonlinear ordinary differential equations. Equations of motion (1) and (2) may be expressed as:

(14) $$\left(D\nabla^{4}w_{t}+\rho_{t}\frac{\partial^{2}w_{t}}{\partial t^{2}}=\ldots ,\phi \right)=\int_{0}^{2\pi }\int_{0}^{L}\left(D\nabla^{4}w_{t}+\rho_{t}\frac{\partial^{2}w_{t}}{\partial t^{2}}=\ldots \right)\times \phi .$$

Galerkin’s weighting function is obtained from the first derivative of Equation (10) with respect to time.

(15) $$\phi =\sum_{m=1}^{2}\sin(m\pi x/L)\cos(n\theta )+\frac{n^{2}}{2R_{t}}\Omega_{m,n}(t)\sin{\left(m\pi x/L\right)}^{2}.$$

After evaluating the integral in Equation (14), the ordinary differential system with unknown functions $\Omega_{m,n}(t)$ is given as:

(16) $$\frac{d^{2}\Omega_{1,n}(t)}{dt^{2}}+\delta_{1n}^{2}\Omega_{1,n}(t)=0,$$

(17) $$\frac{d^{2}\Omega_{2,n}(t)}{dt^{2}}+\delta_{2n}^{2}\Omega_{2,n}(t)=0,$$

(18) $$\frac{d^{2}\Omega_{3,n}(t)}{dt^{2}}+\frac{\gamma \pi^{2}}{L^{2}\left(\rho_{s}+m_{3n}\right)}\Omega_{3,n}(t)=0,$$

(19) $$\frac{d^{2}\Omega_{4,n}(t)}{dt^{2}}+\frac{4\gamma \pi^{2}}{L^{2}\left(\rho_{s}+m_{4n}\right)}\Omega_{4,n}(t)=0,$$

where

(20) $$\delta_{{}_{1n}}^{2}=\frac{Eh}{\rho_{t}}\left[\frac{1}{12(1-\nu^{2})}{\left(\frac{\pi^{2}h}{L^{2}}+\frac{\sqrt{\varepsilon_{n}}}{R_{t}}\right)}^{2}+\frac{\pi^{4}R_{{}^{t}}^{2}}{{\left(\pi^{2}R_{{}^{t}}^{2}+n^{2}L^{2}\right)}^{2}}\right],$$

(21) $$\delta_{{}_{2n}}^{2}=\frac{Eh}{\rho_{t}}\left[\frac{1}{12(1-\nu^{2})}{\left(\frac{4\pi^{2}h}{L^{2}}+\frac{\sqrt{\varepsilon_{n}}}{R_{t}}\right)}^{2}+\frac{16\pi^{4}R_{{}^{t}}^{2}}{{\left(4\pi^{2}R_{{}^{t}}^{2}+n^{2}L^{2}\right)}^{2}}\right],$$

(22) $$m_{3n}=\frac{\rho_{s}LI_{n}(\frac{\pi R_{s}}{L})}{\pi {I^{\prime }}_{n}(\frac{\pi R_{s}}{L})},$$

(23) $$m_{4n}=\frac{\rho_{s}LI_{n}(\frac{2\pi R_{s}}{L})}{\pi {I^{\prime }}_{n}(\frac{2\pi R_{s}}{L})},$$

where $\varepsilon_{n}={\left(\frac{n^{2}h}{R_{t}}\right)}^{2}$ is the nonlinearity parameter (for $\varepsilon_{n}=0$, the vibrations become linear).

### 3.2. The Method of Averaging

The non-linear ordinary differential Equations (16)–(19) can be solved approximately by using the method of averaging [36]. This method is used to obtain simpler relationships between the first and second order derivatives of a function $\Omega_{m,n}(t)$ with a slowly varying amplitude $U_{m,n}(t)$ and phase β(t).

(24) $$\Omega_{m,n}(t)=U_{m,n}(t)\cos(\omega t+\beta (t)).$$

(25) $$∴\frac{d\Omega_{m,n}(t)}{dt}=-\omega U_{m,n}(t)\sin(\omega t+\beta (t))+\frac{dU_{m,n}(t)}{dt}\cos(\omega t+\beta (t))-U_{m,n}(t)\frac{d\beta (t)}{dt}\sin(\omega t+\beta (t)).$$

By applying the assumptions that steady state vibrations and $U_{m,n}(t)$, β(t) are slowly varying functions of time, we get

(26) $$\frac{dU_{m,n}(t)}{dt}\cos\;(\omega \;t+\beta (t))-U_{m,n}(t)\frac{d\beta (t)}{dt}\sin\;(\omega \;t+\beta (t))=0.$$

(27) $$\frac{d\Omega_{m,n}(t)}{dt}=-\omega \;U_{m,n}(t)\sin(\omega t+\beta (t)),$$

Then

(28) $$\frac{d^{2}\Omega_{m,n}(t)}{dt^{2}}=-\omega^{2}U_{m,n}(t)\cos(\omega t+\beta (t))=-\omega^{2}\;\Omega_{m,n}(t).$$

By substituting (26–28) into Equations (16–19), we get

(29) $$\omega_{{}_{1n}}^{2}=\delta_{{}_{1n}}^{2}=\frac{Eh}{\rho_{t}}\left[\frac{1}{12(1-\nu^{2})}{\left(\frac{\pi^{2}h}{L^{2}}+\frac{\sqrt{\varepsilon_{n}}}{R_{t}}\right)}^{2}+\frac{\pi^{4}R_{{}^{t}}^{2}}{{\left(\pi^{2}R_{{}^{t}}^{2}+n^{2}L^{2}\right)}^{2}}\right],$$

(30) $$\omega_{{}_{2n}}^{2}=\delta_{{}_{2n}}^{2}=\frac{Eh}{\rho_{t}}\left[\frac{1}{12(1-\nu^{2})}{\left(\frac{4\pi^{2}h}{L^{2}}+\frac{\sqrt{\varepsilon_{n}}}{R_{t}}\right)}^{2}+\frac{16\pi^{4}R_{{}^{t}}^{2}}{{\left(4\pi^{2}R_{{}^{t}}^{2}+n^{2}L^{2}\right)}^{2}}\right],$$

(31) $$\omega^{2}{}_{3,n}=\frac{\gamma \pi^{2}}{L^{2}\left(\rho_{s}+m_{3n}\right)},$$

(32) $$\omega^{2}{}_{4,n}=\frac{4\gamma \pi^{2}}{L^{2}\left(\rho_{s}+m_{3n}\right)}.$$

## 4. Numerical Simulation Procedure

Equations (29) and (30) were used to evaluate the first and second modes of the nonlinear frequency of a SWCNT, which has been modeled by Donnell’s nonlinear model. For simplicity, it is assumed that SWCNTs are geometrically and physically identical and the numerical calculation has been done for Equations (29) and (30) using the geometries of SWCNTs (Table 1 and Table 2) that were reported by Gupta et al. [37].

In this section, the effects of the nonlinear parameter $\varepsilon_{n}={\left(\frac{n^{2}h}{R_{t}}\right)}^{2}$ on the first and second mode of the frequency are studied.

**Figure 2:**
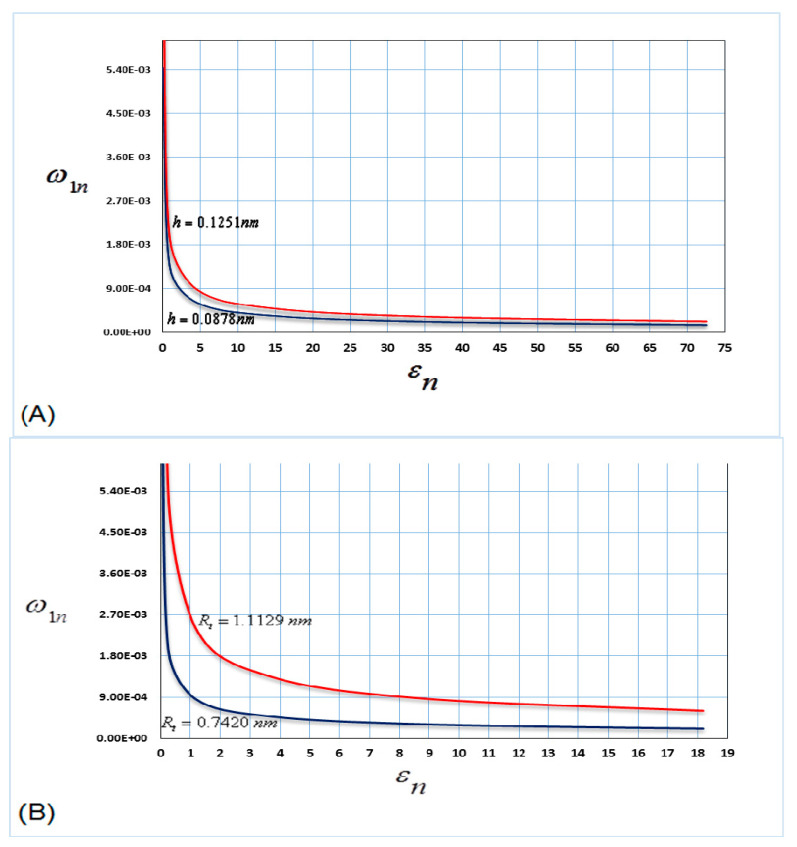
Nonlinear parameters versus frequency for the first mode of vibrations (**A**) h=0.0878 nm, h=0.1251 nm and (**B**) $R_{t}=0.7420\;\mathrm{nm}$, $R_{t}=1.1129\;\mathrm{nm}$.

Figure 2 shows the influence of the nonlinear parameters (radius and thickness of nanotube) on the frequency of the first mode of vibrations, when the nanotube is filled with water.

Figure 2A shows the variation in the vibration frequency for the different values of the nanotube thickness (*h*). The results show that the effects of the nanotube thicknesses are notable at low vibration frequency.

Figure 2B shows the variation in the vibration frequency for the different values of the nanotube radius (*R*). The figure shows that the nonlinear frequency grew with the increasing nanotube radius (*R*). From this figure, it is also clear that the small change in the nanotube radius corresponds to a notable change in the vibration frequency.

**Figure 3:**
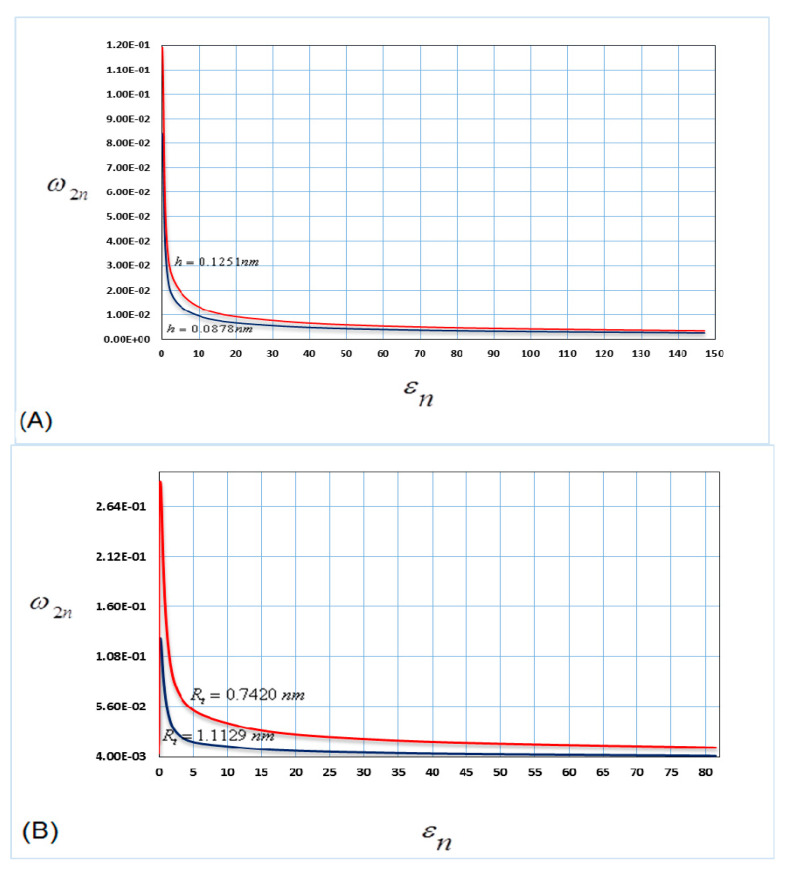
Nonlinear parameters versus frequency for the second mode of vibrations (**A**) h=0.0878 nm, h=0.1251 nm and (**B**) $R_{t}=0.7420\;\mathrm{nm}$, $R_{t}=1.1129\;\mathrm{nm}$.

Figure 3 shows the variation in the nonlinear parameters (radius and thickness of nanotube) versus the frequency for the second mode of vibrations, when the nanotube is filled with water.

Figure 3A shows the variation in the vibration frequency for the different values of the nanotube thickness (*h*). The results show that the nonlinear frequency grew slowly with the change in the nanotube thickness compared with the case of the first mode of vibration.

Figure 3B shows the variation in the vibration frequency for the different values of the nanotube radius (*R*). From this figure, it is clear that the small change in the nanotube radius corresponds to a small increase in the vibration frequency compared with the same case of the first mode of vibrations.

From Figure 2 and Figure 3, the results show that as the nonlinear parameters (radius and thickness of nanotube) increase, the vibration frequency increases. In addition, it is shown that the influence of the nonlinear parameters is greater at the lower mode in comparison with the higher mode for the same thickness and radius of the nanotube.

## 5. Conclusions

In this paper, the nonlinear vibration of pre-stressed fluid-filled single-walled carbon nanotubes with simply supported ends is investigated based on von Karman’s geometric nonlinearity and Donnell’s simplified shell model and the effects of the different nanotube thicknesses and radiuses on the nonlinear vibration frequencies have been discussed in detail. Galerkin’s procedure was used to discretize the governing partial differential equations into ordinary differential equations of motion. A nonlinear analytical formula was obtained for the model and the effects of internal fluid on the vibration of single-walled carbon nanotubes with the different nonlinear parameters have been discussed. As a case study, the mechanical and dimensional properties of the SWCNT were obtained from Gupta et al. [37]. The results show that as the nonlinear parameters (radius and thickness of nanotube) increase, the vibration frequency increases. In addition, it is shown that the influence of the nonlinear parameters is greater at the lower mode in comparison with the higher mode for the same thickness and radius of the nanotube.

## Figures and Tables

**Table 1:** Simulation parameters.

Young’s Modulus E(Gpa)	Mass Density $\rho_{t}(kgm^{-3})$	Poisson’s Ratio υ	Tube (n,m)	Tube Length L (nm)
1060	2270	0.25	(40,0)	10

**Table 2:** The parameters used for modeling (SWCNT).

Radius of Single-Walled Carbon Nanotube (*R_t_*) (nm)	Nanotube Wall Thickness (*h*) (nm)
0.7420	0.0878
1.1129	0.1340
